# Herpes zoster and the subsequent risk of prostate cancer in an Asian population: A nationwide population-based cohort study

**DOI:** 10.1097/MD.0000000000022441

**Published:** 2020-10-02

**Authors:** Yao-Hsuan Tsao, Chi-Jeng Hsieh, Yung-Shun Juan, Yung-Chin Lee, Jung-Tsung Shen, Hsun-Shuan Wang, Jhen-Hao Jhan, Jiun-Hung Geng

**Affiliations:** a Department of Urology, Kaohsiung Municipal Hsiao-Kang Hospital; b Department of Urology, Kaohsiung Medical University Hospital; c Kaohsiung Medical University, Kaohsiung; d Department of Health Care Administration, Oriental Institute of Technology, New Taipei City; e Department of Urology, Kaohsiung Municipal Ta-Tung Hospital, Taiwan.

**Keywords:** age, cancer risk, herpes zoster, immunosuppression, oncology, prostate cancer

## Abstract

It has been suggested that herpes zoster may increase the risk of subsequent prostate cancer (PCa). We aimed to assess the risk of PCa following herpes zoster by the population-based follow-up study.

This is a retrospective study and data are from the Taiwan National Health Insurance Research Database (NHIRD). The study cohort comprised all patients with a diagnosis of herpes zoster (International Classification of Diseases, 9th Revision, Clinical Modification code 053.0–053.9) and followed for PCa from 1997 to 2013 (n = 11,376). Subjects younger than 20 years of age were excluded. The match-control cohort was identified from the Registry of Beneficiaries of the NHIRD and randomly selected by matching with the study cohort at a 3:1 ratio based on age (every 5-year span), and year of herpes zoster diagnosis (n = 34,128). We used Cox proportional hazards regression models to estimate the hazard ratios (HRs) for subsequent PCa, after controlling for potential cormobidities.

Men with and without herpes zoster had similar age and comorbidity distributions. Among the 45,504 sampled patients, 1011 (2.22%) developed PCa during the 10 years of follow-up, 276 (2.43%) from the study cohort and 735 (2.15%) from the match-control cohort and the incidence rate was 3.13 and 2.72 per 1000 person years respectively. Patients with herpes zoster were more likely to develop PCa than patients in the match-control cohort (HR = 1.15; 95% confidence interval (CI) = 1.00–1.32, *P* value = .045). After adjusting for age and comorbidities, herpes zoster was associated with a 1.15 increased risk of PCa (adjusted HR = 1.15, 95% CI = 0.99–1.32, *P* value = .054).

Our study indicates that preceding herpes zoster infection is a suggestive risk marker for subsequent PCa after controlling for potential confounders. Further prospective studies are needed to determine the relationship between herpes zoster and PCa.

## 1 Introduction

As of 2012, prostate cancer (PCa) was the second most frequently diagnosed malignancy (around 15% of all male cancer) and the fifth leading cause of cancer death in men worldwide.^[1]^ Established risk factors for PCa include advanced age, black race, a family history of the disease, and certain genetic polymorphisms.^[2]^ However, a complete understanding of the causes of prostate cancer remains elusive^[3]^ and the etiological factors triggering PCa have not yet been fully identified. There is increasing evidence in the literature suggesting that infection may contribute to prostate carcinogenesis.^[4]^ Infection or inflammation, stimulates the production of inflammatory cytokines and reactive oxygen species, leading to increased cellular proliferation and, possibly, to carcinogenesis.^[5]^ One recent study focused on the role of cystitis and urethritis, which might cause prostatic inflammation, thereby increasing the risk of subsequent PCa.^[6]^

Herpes zoster, also known as shingles, is caused by the reactivation of the varicella-zoster virus previously latent in the sensory ganglia and dorsal nerve roots, and it is characterized by a painful skin rash with blisters in a localized area.^[7]^ Typically, it has been associated with a weakened immune system, including old age, severe physical injury, patients receiving chemotherapy, and long-term steroids use.^[8]^ As is known, the risk of cancers is also elevated when people are in immuno-suppressed states.^[9]^ Therefore, it is hypothesized that one might have an increased incidence of subsequent cancer, including PCa, within the population of patients with herpes zoster.

Several cohort studies have attempted to determine the association between herpes zoster and the risk of subsequent cancer as shown as Table 1. A paper published by Ragozzine et al showed no significant association in the incidence of subsequent cancer between herpes zoster patients and controls (95% Confidence Interval (CI), 0.9–0 1.3) and the subgroup analysis demonstrated that most of the specific cancer sites were not different except for those for colon and bladder cancers in women.^[10]^ Following this, Buntinx et al enrolled more zoster patients (n = 1211) and found that above the age of 65 years, a significant increase of cancer existed in the group of females (hazard ratio = 2.65 95% CI, 1.43–4.90), but not in males and not in PCa.^[11]^ In 2013, Chiu et al and Cotton et al both reported a significant association between herpes zoster and subsequent cancer for all age groups and both genders. Additionally, the incidence of PCa was higher in males with herpes zoster than in patients without herpes zoster, which was different from the previous study.[^12^,^13^] Recently, Qian et al have reported that prior to hematological cancer diagnosis, the incidence of herpes zoster was higher than those without cancer, but not in solid tumors.^[14]^

**Table 1:**
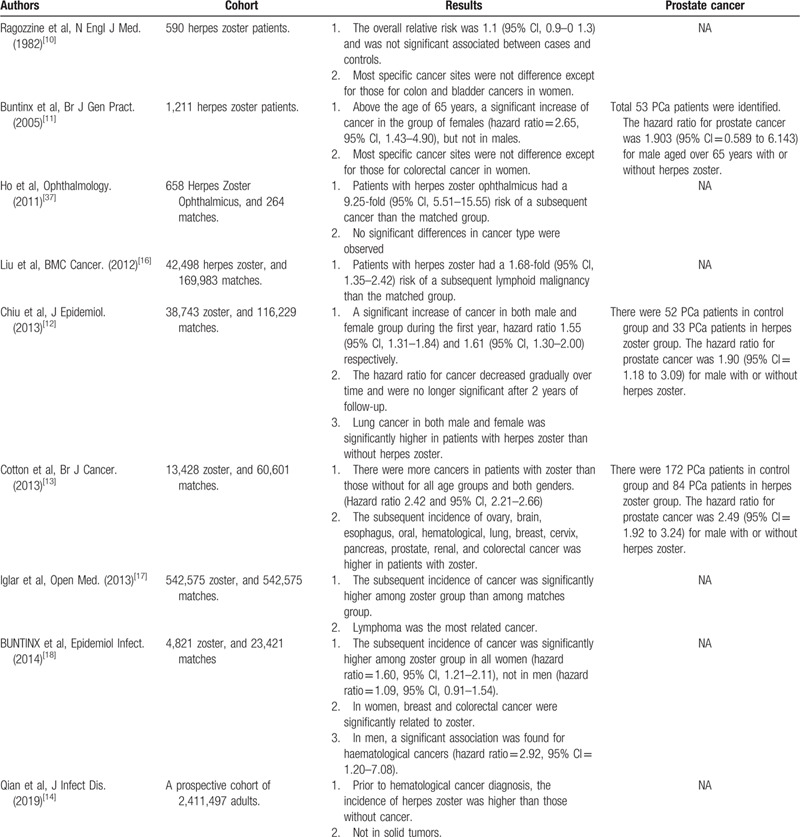
Cohort Studies of Herpes Zoster and the risk of subsequence cancers.

There is still controversy over the association between a previous diagnosis of herpes zoster and the risk of subsequent diagnosis of cancer. Moreover, studies concerning herpes zoster and PCa are inadequate and deficient. As mentioned previously, 3 publications included PCa, but all the numbers of PCa patients were less than300 . Inconsistent results were also noted; 1 showed no significant association and the others showed significant association. Furthermore, all these studies were focused on all cancer sites, not just on PCa.[^10^,^11^,^12^,^13^,^14^,^15^,^16^,^17^,^18^] In order to further assess the relationship between herpes zoster and the risk of subsequent PCa, we conducted this study in a large cohort by using a nationwide population-based dataset from Taiwan.

## 2 Materials & methods

### 2.1 Data source

Data were obtained from the National Health Insurance Research Database (NHIRD), which was set up and managed by the Taiwan National Health Research Institute (NHI). Taiwan NHI is a single-payer compulsory social insurance plan that centralizes the disbursement of healthcare funds. The system promises equal access to healthcare for all citizens, and the population coverage reaches 99% of the countrys population (23.75 million citizens).[^15^,^19^] The Taiwan NHI is continuously and prospectively registering all new diagnoses together with new drug prescriptions and personal information from a wide range of health care services, including outpatient visits, inpatient care, dental care, traditional Chinese medicine, renal dialysis, and prescription pharmacy.^[20]^ The database is a national, population-based claims database, and all personally identifiable information is encrypted for patient protection. Due to the huge, comprehensive and fully-fledged data collection, many researchers have published studies based on this data.^[21]^

The data of our study population were gained from the Longitudinal Health Insurance Database 2005 (LHID 2005), a subset of the NHIRD.^[22]^ The LHID 2005 consists of all the original claim data of medical records of 1,000,000 individuals randomly sampled from Registry for Beneficiaries of the NHIRD, which maintains the registration data of everyone who was a beneficiary of the NHI program during the period of 1997 to 2013. No significant difference in gender distribution was noted between patients in the LHID2005 and the original NHIRD.^[23]^ The diagnoses in the LHID 2005 are coded using the International Classification of Diseases, Ninth Revision, Clinical Modification (ICD-9-CM).

### 2.2 Ethics statement

The NHIRD encrypts patient personal information to protect privacy and provides researchers with anonymous identification numbers associated with relevant claims information, including sex, date of birth, medical services received, and prescriptions. Therefore, patient consent is not required to access the NHIRD. This study was approved to fulfill the condition for exemption by the Institutional Review Board (IRB) of Kaohsiung Medical University Hospital (KMUHIRB-E(II)-20190362). The IRB also specifically waived the consent requirement.

### 2.3 Sampled participants

This retrospective study contained a study cohort and a match-control cohort for comparison. From the dataset, we identified all male patients aged 20 years or older who received ambulatory care for herpes zoster (the ICD-9-CM diagnostic code 053.0–053.9) from 1997 to 2013 as the study cohort (n = 11,376). The date of the first medical visit of a herpes zoster patient registered was defined as the index date. The matched-control cohort was identified from the Registry of Beneficiaries of the NHIRD and randomly selected from the LHID 2005 matching with the study cohort at a 3:1 ratio based on age (every 5-year span), and year of herpes zoster diagnosis (n = 34,128). Patients with a history of prostate cancer before the index date were excluded. Subjects were followed-up until diagnosis of PCa (ICD-9-CM code 185), death, withdrawal from the NHI program, or December 31, 2013.

### 2.4 Statistical analysis

All men were followed from the date of diagnosis until death, emigration or end of follow-up (December 31, 2013), whichever event came first. We accessed the χ2 or Fishers exact test to evaluate the differences between categorical parameters. Basic social demographic data such as age, insurance range, and selected comorbidities were considered risk factors for the diagnosis of PCa. We included diabetes mellitus (DM),^[24]^ hyperlipidemia,^[25]^ hypertension,^[26]^ cardiovascular disease,^[27]^ cerebral vascular disease,^[28]^ and peripheral occlusive arterial disease^[29]^ as selected comorbidities due to the potential risk of cancer and these potential confounders were adjusted in our study. Log-rank tests were used to evaluate the difference in the risk of subsequent PCa between these 2 cohorts. Cox proportional hazard regressions were conducted to calculate hazard ratios (HRs) and 95% CIs for subsequent PCa, while controlling for the above-mentioned potential confounders. Statistical significance was set at *P* < .05. SPSS 20.0 (SPSS Inc., Chicago, IL) and was used for all statistical analyses.

## 3 Results

This study consisted of 11,376 patients with diagnosis of herpes zoster and 34,128 match-control individuals. Table 2 shows the demographic characteristics and medical conditions of the study subjects. The mean ± (standard deviation) age of subjects with and without herpes zoster was 60.04 ± 12.52 and 60.25 ± 12.42 years old, respectively. As compared with patients without herpes zoster, patients with herpes zoster were more likely to have cerebral vascular disease and other profiles, including diabetes mellitus, hyperlipidemia, hypertension, cardiovascular disease, and peripheral occlusive arterial disease were not significantly different. (Table 2).

**Table 2:**
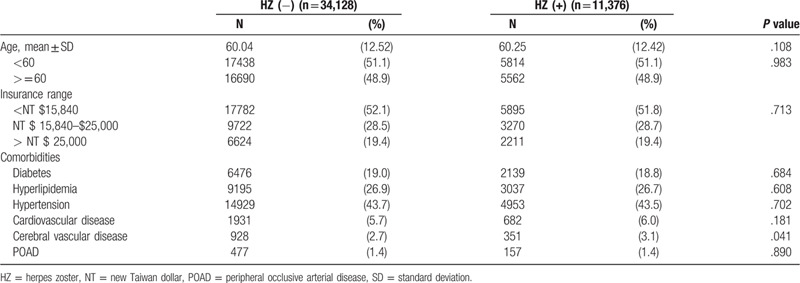
Demographic data (N = 45,504).

Among the 45,504 sampled patients, 1011 (2.22%) developed PCa during the 10 years of follow-up, 276 (2.43%) from the study cohort and 735 (2.15%) from the match-control cohort and the incidence rate was 3.13 and 2.72 per 1000 person years respectively. (Table 3)

**Table 3:**
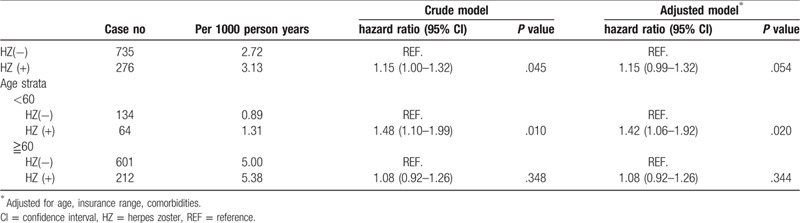
The risk of prostate cancer associated with herpes zoster infection.

**Figure 1:**
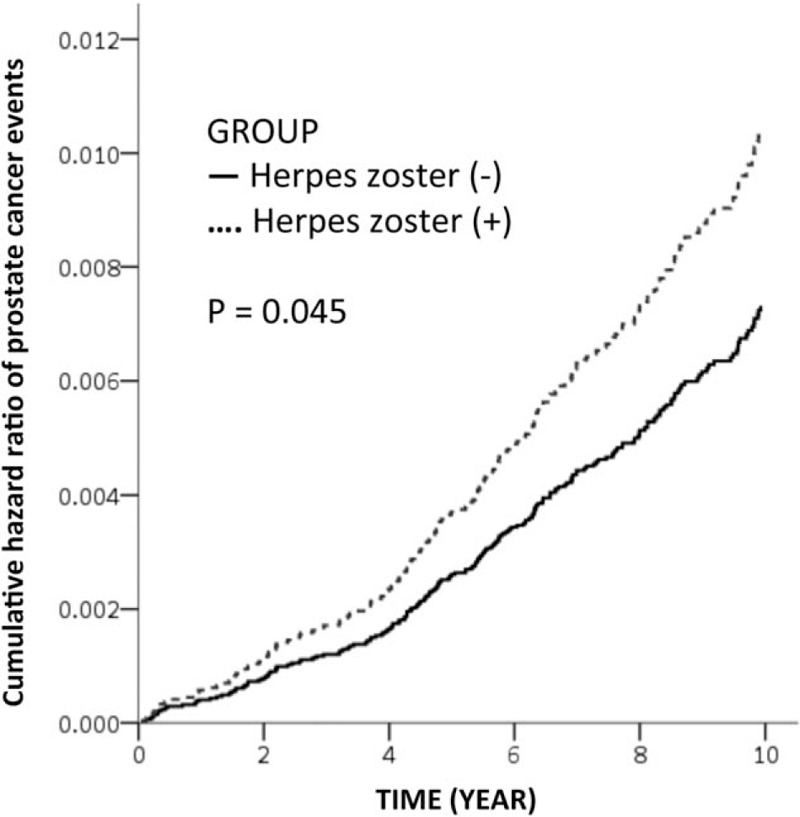
Cumulative hazard ratio of prostate cancer events in subjects with and without herpes zoster.

Patients with herpes zoster were more likely to develop PCa than patients without herpes zoster in the match-control cohort (crude HR = 1.15; 95% CI = 1.00–1.32, *P* value = .045) as shown in Table 3 and Figure 1. After controlling for confounders, men with herpes zoster were suggestively having an increased risk of PCa (HR 1.15, 95% CI = 0.99–1.32, *P* value =.054). This After age stratification, the adjusted HR for subsequent PCa was 1.42 (95% CI = 1.06–1.92) in those aged less than 60 years, which showed significant difference (*P* value = .020).

Table 4 shows the HRs for subsequent PCa according to follow-up period after the index day. The HR was 1.00 (95% CI, 0.58–1.70) for the first year, 1.02 (95% CI, 0.77–1.36) for the 3rd year, 1.09 (95% CI, 0.88–1.34) for the 5th year and 1.15 (95% CI, 0.99–1.32) for the 10th year. An increase in the risk of subsequent PCa was observed in a longer follow-up period after an initial diagnosis of herpes zoster.

**Table 4:**
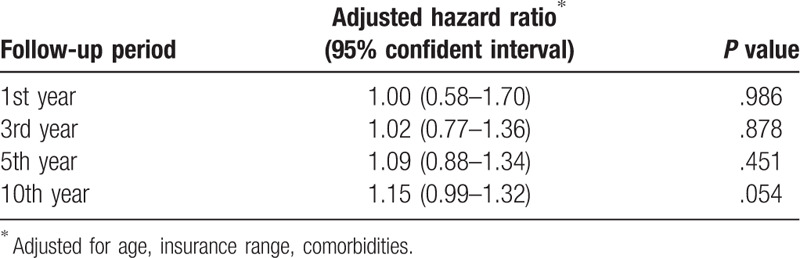
Hazard ratios for prostate cancer after a herpes zoster diagnosis, stratified by follow-up period.

## 4 Discussion

In this population-based matched-control study, we used Taiwanese men to explore the association between herpes zoster and the subsequent risk of PCa. To our knowledge, this is the largest matched-control study focusing on the relationship between herpes zoster and PCa currently (n = 45,504, including subsequent 1011 PCa patients). We found that preceding herpes zoster infection is a suggestive risk marker for subsequent PCa in individuals after controlling for potential confounders (HR = 1.15, 95% CI = 0.99–1.32). After age stratification, herpes zoster was associated with a 1.42-fold increased risk of PCa in those aged less than 60 years (HR: 1.42, 95% CI = 1.06–1.92).

Our findings are similar to the results of the two studies,[^12^,^13^] listed in Table 1, reported that the subsequent incidence of PCa was higher in men with zoster, where hazard ratios were 1.90 (95% CI = 1.18–3.09) and 2.49 (95% CI = 1.92–3.24) respectively. But both of them had smaller PCa populations comparing to our study. Besides, the selected comorbidities as risk factors for PCa were different between our study and theirs. We enrolled as many comorbidities as possible, including diabetes mellitus, hypertension, hyperlipidemia and cardiovascular disease, but on the contrary, one of the other 2 studies chose diabetes mellitus and the other 1 chose smoking as their adjusted factors. Consequently, our study has some advantages, but the relationship between herpes zoster and PCa are still controversy and need further larger and prospective studies to figure it out.

There is another study published on 2005 by Buntinx et al, which showed no significant difference in the subsequent incidence of PCa for male aged over 65 years with or without herpes zoster (HR = 1.903, 95% CI = 0.589–6.143).^[11]^ However, there were only 53 PCa patients in this cohort, which is relatively small. Furthermore, they did not mention about the subsequent incidence of PCa in men younger than 65 years old. We had 1011 PCa patients in our cohort and enrolled much wider age groups. This may be explained the differences between our study and theirs.

Various mechanisms have been proposed to explain the increased risk of PCa following herpes zoster. First, herpes zoster is an indicator of occult malignancy.^[30]^ It is suggested that herpes zoster could be an early manifestation of immune impairment associated with occult malignancy. Second, the herpes zoster may weaken the immune system which allows tumor cells to escape from immune surveillance.[^31^,^32^] Third, the zoster virus may induce carcinogenesis by chronic inflammation and alteration of oncogenes or tumor suppressors.^[33]^ In the present study, an increased risk of PCa long after an herpes zoster diagnosis was noted. Compared with the first year and the first 5 years after the diagnosis of herpes zoster, the risk of PCa in the period of 10 years follow-up showed higher. The results do not support the mechanism of herpes zoster being an indicator of occult malignancy. Rather, the higher HRs for subsequent PCa during a longer follow-up may reflect an underlying immune dysfunction would increase the persons risk of PCa or direct carcinogenesis after herpes zoster diagnosis. However, these findings need to be validated by a larger and prospective study.

The present study has several strengths. There is a large sample size and a well-defined population. In addition, we focused on the relationship between PCa and herpes zoster, which enhances the understanding of both diseases. We also stratified our study population by age and found differences between age groups. Finally, we analyzed the risk of PCa by time since start of follow-up to clarify the possible mechanisms. Nevertheless, we also have limitations in our study and the results of our study should be interpreted in the context of these limitations. Firstly, the coding system relies on physician-reported diagnoses, and there are potential biases in miscoding and error diagnosis. However, the possibility of miscoding and error diagnosis is not likely different between study cohort and matched-control groups, and both herpes zoster and PCa have unique clinical presentations. Secondly, although we enrolled as many as possible confounders, there are some confounding factors that were not included in our study, such as smoking status. Thirdly, our population is of Han Chinese ethnicity, which may not be the same as other ethnic groups. Fourthly, we did not use propensity score in our study to reduce selection bias,^[34]^ especially for diabetes mellitus, which might be related to immunocompromised conditions. Instead, we randomly selected the match-control cohort by matching age and year of herpes zoster diagnosis. Furthermore, there was no difference in the proportion of diabetes mellitus between the study cohort and matched-control cohort in present study. This could minimize the selection bias. Fifthly, we did not use competing risks methods^[35]^ in our study, which might overestimate the risk of PCa by failing to account for the competing risk of death. Based on the data from Centers for Disease Control and Prevention (CDC), 96 deaths happen per year due to herpes zoster (less than 1 per 1 million population),^[36]^ which means the competing risk of death for herpes zoster is small. Due to the small number of death, it could minimize the overestimation of the risk of PCa. Finally, the censoring in this dataset should be considered because some patients with herpes zoster may not have visited a doctor. However, access to primary physician doctors in Taiwan is excellent as we have possibly the most convenient and economical National Health Insurance Scheme (national government system) in the world. Therefore, we believe that most patients with a painful skin rash and blisters would seek medical care after disease onset and the censoring data might be small.^[37]^ Finally, we did not collect the varicella vaccination status in our cohort, which may be more interesting in the field of public health. Further studies could add the variable and try to understand whether varicella vaccination could affect the incidence of subsequent PCa or not.

## 5 Conclusions

Our study indicates that preceding herpes zoster infection is a suggestive risk marker for subsequent PCa after controlling for potential confounders. Further prospective studies are needed to determine the relationship between herpes zoster and PCa.

## Author contributions

**Conceptualization:** Jiun-Hung Geng, Yao-Hsuan Tsao.

**Data curation:** Jhen-Hao Jhan, Jiun-Hung Geng.

**Formal analysis:** Chi-Jeng Hsieh, Jiun-Hung Geng, Yao-Hsuan Tsao.

**Funding acquisition:** Hsun-Shuan Wang, Yung-Chin Lee, Yung-Shun Juan, Jung-Tsung Shen

**Investigation:** Jiun-Hung Geng, Yung-Chin Lee.

**Methodology:** Chi-Jeng Hsieh, Jiun-Hung Geng, Jhen-Hao Jhan.

**Project administration:** Yung-Shun Juan, Jiun-Hung Geng.

**Resources:** Jung-Tsung Shen.

**Supervision:** Jung-Tsung Shen, Hsun-Shuan Wang, Jiun-Hung Geng.

**Writing–original draft:** Yao-Hsuan Tsao.

**Writing–review & editing:** Jiun-Hung Geng.
